# Market choices driven by reference groups. An evolutionary approach

**DOI:** 10.1007/s00191-015-0402-z

**Published:** 2015-04-01

**Authors:** Michał Ramsza

**Affiliations:** Department of Mathematics and Mathematical Economics, Warsaw School of Economics, Al. Niepodłeglości 162, Warsaw, 02-554 Poland

**Keywords:** Reference groups influence, Population games, Learning, C73, D71, D83

## Abstract

The present paper tries to answer analytically how much the reference group influence can affect an actual market share of a particular brand or product. It is found that the increase of the size of a reference group and the probability of following the majority within the reference group may lead to the temporary modest prevalence of one brand. This result requires a relatively large size of a reference group and a high probability of following the majority within the reference group. If these conditions are not satisfied the effect on the market share is negligible.

## Introduction

The concept of a reference group and its potential influence on an individual has been know at least since the landmark publication of Merton (1949), but its beginnings can be traced to Cooley (1902). A brief definition of a reference group is given in Park and Lessig (1977):

“(...) a reference group is defined to be an actual or imaginary individual or group conceived of having significant relevance upon an individual’s evaluations, aspirations, or behavior.”

The present paper uses the concept of a reference group in a very broad sense as defined above. The general discussion regarding the concept of a reference group is beyond the scope of this paper. A brief discussion of the history of the concept of a reference group and its relevance can be found in Dawson and Chatman (2001).

This short note concerns the choices between two alternatives made within a large population. Suppose that each individual in the population uses a product of one of two alternative brands. Once in a while the product breaks and needs to be replaced with a new one. At this time, an individual tries to determine which brand is used by the majority of a population. He selects at random a group of $k \in \mathbb {N}$ individuals. This group becomes his reference group. He chooses a brand used by the majority of those in the reference group with the probability *α*∈(0,1), referred to as the follow-up probability. This procedure is repeated in time and leads to fluctuations of the share of each brand in the market. The market share of the first brand at any given time *t* is denoted by *x*(*t*) ∈[0,1].

The two parameters, the size of a reference group *k* and the follow-up probability *α*, are exogenous. The size *k* of the reference group governs the precision of information that an individual gets while choosing a brand.^1^ The follow-up probability *α* is the measure of affect’s strength of an individual for the group. The strength of the influence of the group on the individual thus measures how strongly an individual wants to be associated with the majority of a population. The value *α* = 1/2 is referred to as the neutral position — larger values indicate the positive attitude to the information, while lower values indicate the negative attitude to the information.

The main issue investigated in the present paper is the *isolated effect of the reference groups on the market share of a particular brand*. Specifically, there are two main points investigated. The first point concerns the existence and (local) stability of equilibrium. The second point concerns the dependence of equilibrium on the follow-up probability *α* and the size of a reference group *k*. It is shown that, depending on the follow-up probability, there are two distinct types of model behavior, i.e. the model is not structurally stable for any *k*. The (pitchfork) bifurcation occurs for relatively high values of the follow-up probability provided that the size of a reference group is small, which may be interpreted as the negligible effect of the reference group in certain circumstances.

The rest of the paper is organized in the following way. In Section 2, the model is constructed and analyzed. Section 3 offers discussion and conclusions.

## The model and analysis

### The model

A large but finite population of a size $ N \in \mathbb {N} $ of individuals is assumed. Each individual may choose one of two alternatives (brands). At each period *t* = 0, *δ*,2*δ*,…, where *δ*>0, there is a fraction of the population using the first brand. This fraction, as mentioned above, is denoted by *x*(*t*) . The share of a population using the other brand is 1−*x*(*t*).

The learning algorithm employed by members of the population is very simple. At each period *t*, a single individual is selected at random.^2^ This individual randomly chooses a group of *k* persons from the population, referred to as the reference group.^3^ An individual in question determines which alternative is in the majority in a reference group and chooses this alternative with the probability *α*∈(0,1). The probability of following the majority in a reference group is called the follow-up probability. This process is repeated during every period.

In the above construction, the time is assumed to be discrete, *t* = 0, *δ*,2*δ*,…, and the size of a population *N* is finite. The state of a population can be described in several different ways, but because the model is concerned only with the market share of each brand, it is described by the share of a population *x*(*t*) using the first brand. Given that the size of a population *N* is finite, the set of possible states of a population {0,1/*N*,2/*N*,…,(*N*−1)/*N*,1} is also finite. The procedure of brand selection defines the transition probabilities between the states of a population. These probabilities are denoted by *p*_*ij*_(*x*), that is, *p*_*ij*_(*x*) is the probability that a single individual changes the brand from the *i*-th alternative to the *j*-th alternative, *i*, *j* = 1,2, given the state of a population *x*.

The above model is, in fact, a finite Markov chain. This Markov chain can be studied directly, but it is easier to consider a system of differential equations of the form

1 $$ \left\{ \begin{array}{llll} \hspace*{1.5pc} \dot{x} &=& p_{21}(x) - p_{12}(x), \\ 1-\dot{x} &=& p_{12}(x) - p_{21}(x). \end{array} \right. $$

System (1) approximates^4^ the average behavior of the Markov chain; cf. (Benaim and Weibull 2003). The induced system of differential Eqs. (1) is derived by taking the limit *N* → *∞* with *δ* = 1/*N* but for the approximation to hold, it is enough to have the size *N* of a population finite and large enough.

The size *k* of a reference group defines, together with the follow-up probability *α*, the transition probabilities and is not related in any way to the size of a population but only to the state of a population, that is, as long as the state of a population is *x* and both *k* and *α* are fixed, then the size *N* of a population can be arbitrary. It is assumed only that *N* is larger than *k*. In fact, in the context of the background story, it is better to think of *k* as a much smaller number than the size *N* of a population.

It is clear that, for two alternatives, one of the equations in Eq. 1 can be dropped and from now on it is assumed that the second equation is dropped and only the following equation is used

2 $$ \dot{x} = p_{21}(x) - p_{12}(x). $$

The intuition behind the Eq. 2 is that the change in the share of a population supporting the first alternative is the difference between the rate of inflow into the first alternative and the outflow from it. The approximation (2) is considered in continuous time and solutions take values in the interval [0,1]. However, for large *N*, the grid of states of the Markov chain is very dense and, with a slight abuse of notation, the same notation is used for both.^5^

### Small reference group

For any given *k*, it is simple to calculate the probabilities *p*_*ij*_(*x*). However, for simplicity, it is assumed^6^in this section that *k* = 3. With this assumption the probability *p*_12_(*x*) reads

3 $$\begin{array}{@{}rcl@{}} p_{12}(x) &=& x \left( \alpha \left((1-x)^{3} + 3 (1-x)^{2} x\right)\right.\\ &&\left. + (1-\alpha) \left(x^{3} + 3 (1-x) x^{2}\right)\right) \end{array} $$

and the probability *p*_21_(*x*) reads

4 $$\begin{array}{@{}rcl@{}} p_{21}(x) &=& (1-x) \left( \alpha \left(x^{3} + 3 x^{2} (1-x)\right)\right.\\ &&\left. +(1-\alpha) \left((1-x)^{3} + 3 (1-x)^{2} x\right)\right). \end{array} $$

The probability *p*_12_ is the product of two probabilities. They are the probability that the supporter of the first alternative is selected and the conditional probability that the second alternative is followed. The probability that the supporter of the first alternative is selected, given the state *x* of a population, is simply *x*. The conditional probability that the second alternative is followed is the sum of probabilities of four events: every individual in the reference group uses the second alternative and the majority follows what happens with the probability *α*(1−*x*)^3^; two individuals in the reference group use the second alternative and the majority follows with the probability *α*3(1−*x*)^2^ *x*; only one individual in the reference group uses the second alternative and the minority follows with the probability (1−*α*)3(1−*x*)*x* ^2^; and finally all individuals in the reference group use the first alternative and the minority follows with the probability (1−*α*)(1−*x*)^3^.

Symmetrically, the probability *p*_21_ is also the product of two probabilities. They are the probability that the supporter of the second alternative is selected and the conditional probability that the first alternative is followed. In the considered model, it is assumed that the probability of following a given alternative is independent of the currently supported alternative. Thus, the conditional probability of following a given alternative is the same for both currently supported alternatives, i.e. the probabilities *p*_12_(*x*) and *p*_21_(*x*) differ only by the first term *x* and (1−*x*), respectively, and switching every occurrence of *α* to (1−*α*) and the other way around.

Ultimately, given the above assumptions, the differential equation describing the evolution of the share *x*(*t*) reads

5 $$ \dot{x} = f(x) = (1-2x) \left( x^{2} (2 \alpha -1) - x (2\alpha -1) +1 - \alpha \right). $$

The first thing to notice about Eq. 5 is that the right hand side *f*(*x*) is a polynomial. Thus, for any initial conditions, there is a unique solution defined for *t*≥0. Second, the interval [0,1], which is a projection of the two-dimensional simplex, is forward invariant because *f*(0)=1−*α*>0 and *f*(1)=*α*−1<0.

For any follow-up probability *α*, there is an equilibrium $ \hat {x}^{a} = 1/2 $ and because

$$\frac{df(\hat{x}^{a})}{dx} = \frac{6}{2} \left( \alpha - \frac{5}{6} \right) $$

this equilibrium is stable for *α* < 5/6 and unstable for *α*>5/6.

For *α*>5/6, there are two additional equilibria, namely

$$\hat{x}^{b} = \frac{1}{2} - \sqrt{\frac{5-6\alpha}{4 (1-2 \alpha)}} \quad \text{and} \quad \hat{x}^{c} = \frac{1}{2} + \sqrt{\frac{5-6\alpha}{4 (1-2 \alpha)}}. $$

Both equilibria are stable for *α*>5/6 because

$$\frac{df(\hat{x}^{b})}{dx} = \frac{df(\hat{x}^{c})}{dx} = 5-6\alpha < 0 \quad \text{for}~~\alpha > \frac{5}{6} . $$

**Fig. 1:**
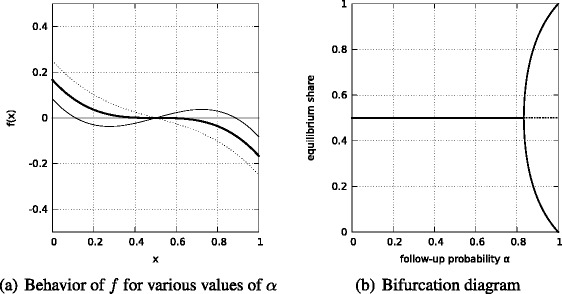
**a** Behavior of *f* for various values of the follow-up probability *α*. The *dotted line* is for *α* = 9/12, the *solid bold line* is for *α* = 5/6 and the *thin solid line* is for *α* = 11/12. **b** Bifurcation diagram for (5). *Solid lines* are for the stable equilibriums, the *dotted line* is for the unstable equilibrium

At *α*_0_ = 5/6 a pitchfork bifurcation occurs.^7^ Figure 1 shows the behavior of *f* for various values of the follow-up probability *α* and the bifurcation diagram for (5).

### Large reference group

The analysis in the previous section is done for a small reference group where *k* = 3. The obvious question is what happens for larger values of *k*>3. It is possible to derive exact formulas for *p*_*ij*_ for any fixed *k*. However, the polynomials describing the probabilities of transitions become large and unwieldy very quickly. Therefore, it is easier to approximate these probabilities by the central limit theorem.

Let *l* be the number of people supporting the first alternative in a randomly selected reference group of a size *k*; *l* is a random variable following the binomial distribution with parameters *k* (a number of trials); and *x* is the probability of a success. Let *w* denote the probability that *l*>*k*/2, that is, *w* is the probability that the majority of individuals in the selected reference group supports the first alternative given the state of a population *x*. For large values of *k*, the probability *w* is approximated by the standard normal cumulative distribution function Φ as follows

6 $$\begin{array}{@{}rcl@{}} w &=& \text{Pr}\left[{l> \frac{k}{2}}\right] = 1 - \text{Pr}\left[{l \leq \frac{k}{2}}\right]\\[-1pt] &=& 1 - \text{Pr}\left[{l - k x \leq \frac{k}{2} - kx}\right]\\[-1pt] &=& 1 - \text{Pr}\left[{\frac{l - k x}{\sqrt{k x (1-x)}} \leq \frac{k/2 - kx}{\sqrt{k x (1-x)}}}\right]\\[-1pt] &\approx &1 - {\Phi}\left(\frac{k/2 - kx}{\sqrt{k x (1-x)}}\right)\\[-1pt] &=& 1 - \frac{1}{2} \left[1 + \text{erf}\left({\frac{k/2 - kx}{\sqrt{2 k x (1-x)}}}\right) \right], \end{array} $$

where erf() is the standard error function, given as

$$\text{erf}({x}) = \frac{2}{\sqrt{\pi}} {\int\limits_{0}^{x}} \mathrm{e}^{-\tau^{2}} d\tau $$

and the approximation follows by the central limit theorem.

The transition probabilities are

7 $$\begin{array}{@{}rcl@{}} p_{12}(x) &=& x (\alpha (1-w) + (1-\alpha) w)\\ p_{21}(x) &=& (1-x) (\alpha w + (1-\alpha) (1-w)). \end{array} $$

Taking into account (2), (6) and (7) the equation analogous to Eq. 5 reads

8 $$ \dot{x} = g(x) = \left(\frac{1}{2}-x\right) + \left(\alpha - \frac{1}{2} \right) \text{erf}\left({\frac{\sqrt{k}}{2 \sqrt{2}} \; \frac{2 x -1}{\sqrt{x (1-x)}}}\right). $$

The right hand side of Eq. 8 is not defined for *x* = 0 or *x* = 1. However, the limits at these points are

$$\lim\limits_{x \to 0^{+}} g(x) = 1-\alpha >0 \qquad\text{and}\qquad \lim\limits_{x \to 1^{-}} g(x) = \alpha-1 <0. $$

These limits are taken as the values of *g*(*x*) for *x* = 0 and *x* = 1. Since *g*(0)>0 and *g*(1)<0, the interval [0,1] is forward invariant under *g*. Also, *g* is a *C* ^1^ function. Thus, for any initial condition, there exists a unique solution defined for *t*≥0.

For any *α*∈(0,1), there is an equilibrium $ \hat {x}^{a} = 1/2 $ because erf(0)=0. The derivative at *x* = 1/2 reads

$$\frac{d g(x)}{d x} = \frac{(2 \alpha -1) \sqrt{k} \mathrm{e}^{\frac{k (1-2 x)^{2}}{8 (x-1) x}}}{4 \sqrt{2 \pi } (-(x-1) x)^{3/2}}-1 \quad \text{and} \quad \frac{d g(1/2)}{d x} = \sqrt{\frac{2}{\pi }} (2 \alpha -1) \sqrt{k}-1. $$

Further, *dg*(1/2)/*dx* = 0 for

$$\alpha_{0} = \frac{1}{4} \left(\frac{\sqrt{2 \pi }}{\sqrt{k}}+2\right). $$

For *α* < *α*_0_, the equilibrium $ \hat {x}^{a} $ is stable and, for *α*>*α*_0_, the equilibrium $ \hat {x}^{a} $ is unstable. At *α*_0_, the pitchfork bifurcation occurs.^8^

**Fig. 2:**
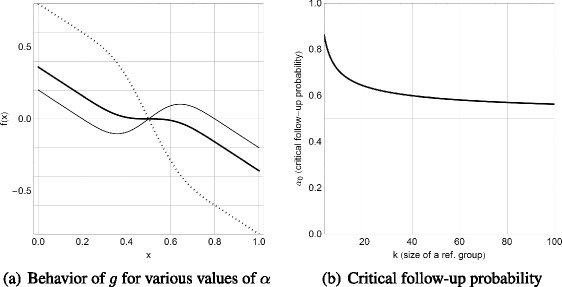
**a** Behavior of *g* for various values of the follow-up probability *α*. The *dotted line* is for *α* = 2/10, the *solid bold line* is for *α* = *α*_0_ and the *thin solid line* is for *α* = 8/10. All plots for *k* = 20. **b** Behavior of the critical follow-up probability *α*_0_ with respect to the cardinality of the reference group *k*

The behavior of a model for large reference groups is qualitatively identical to the simple model with *k* = 3. However, the particular value of the critical follow-up probability *α*_0_ depends crucially on the size of a reference group. The larger the reference group, the smaller the critical follow-up probability *α*_0_. Eventually, for *k* → *∞*, the critical follow-up probability would converge^9^ to 1/2. Figure 2a shows the behavior of *g* for various values of *α* and *k* = 20. Figure 2b shows the dependence of *α*_0_ on the size of a reference group.

## Discussion and conclusions

The presented model is concerned solely with the isolated effect of the assumed algorithm of choosing a brand. As a result, any potential characteristics differentiating the brands are not part of the model and, consequently, the results are symmetric about the equal split of a market between two brands. The results of a model should be interpreted as the potential edge that one brand can have due only to the reference group influence and not the actual preferences of population’s members, since those are absent from the model. The main result of the model is that this potential edge should be relatively small if the size of a reference group is small and the follow-up probability is not too large. This behavior results from the imprecise information from the reference group and is amplified if an individual is not certain how to act upon the information.

It is very important to remember that the differential Eqs. 5 and 8, for *k* = 3 and *k*>3, respectively, are only approximations to the actual Markov chain. It is clear that the Markov chain approximated by the Eqs. 5 or 8 is ergodic for any *α*∈(0,1) and so there is a unique stationary distribution depending on *k* and *α*. This stationary distribution is symmetric about *x* = 1/2. For *α* < *α*_0_, the stationary distribution is a single peak distribution. For *α*>*α*_0_, the stationary distribution becomes a bimodal distribution with peaks aligned with the stable equilibria of a differential equation. As the value of a follow-up probability *α*→1, these peaks converge to 0 and 1. For *α* = 1, these states of the Markov chain are absorbing.

The interpretation of these results is the following. The market share of the first brand fluctuates around *x* = 1/2 as long as *α* < *α*_0_, with increasing variance for *α* → *α*_0_. Once this critical value of *α* is crossed, the behavior changes. Any realization of the Markov chain spends most of the time near the stable equilibria of a differential equation, with occasional switches between them. The time spent near one of the equilibria depends on the size *N* of a population. The larger is *N*, the longer the time^10^ spent near the equilibrium before switching to the other equilibrium.

The setup of the presented model is somewhat similar to other models, in particular to the models where the population is identified with the set of nodes of a graph and the reference group of a player (node) is then identified with the set of its neighbors, as in DeGroot (1974), Watts (2002) or Acemoglu et al. (2011). The present model also can be fitted within this general setup, provided that the graph is the Poisson graph^11^ with the average degree equal to the size of a reference group. In this setting, Eqs. 5 and 8 are usually referred to as the mean-field equations or the master equation; cf. (Helbing 1995).

There are two modeling choices made in the present paper. The first concerns the random choice of a reference group. This is the typical choice for evolutionary game theory, population learning theory and other similar models such as the ones mentioned before. It allows for rigorous analytical analysis of the model. The compromise is the structure of a network of connections and the type of results (average behavior) that can be achieved. The random choice procedure used in the model can be thought of as reflecting an individual observing other randomly selected people and choosing a brand accordingly.

The second modeling choice concerns the follow-up probability. There are other choices possible, e.g. the threshold rule used in Watts (2002), best-reply type rules or rules based on imitations. All these rules have one thing in common: they do not allow for innovations in the sense that, if all members of a population use the same brand, then every individual making the switch decision chooses the brand used by all members of a population.^12^ In terms of the Markov chain, it means that these two states (everyone uses the first brand and everyone uses the second brand) are absorbing (and consequently asymptotically stable equilibria).

The concept of a reference group is used in the present paper in a very broad sense as introduced in Section 1. The speculations on the precise meaning of the presented model for theoretical sociology and economics is left for future research. It is, however, clear that, in the light of recent papers such as Kramer et al. (2014), it is important to try to understand if it is possible to construct and to provide a product that, through the manipulation of the follow-up probability, can swing the brand shares in favor of a particular brand.

Ultimately, the main insight from the presented model is the interplay between the precision of information and the strength of reaction to this information. If the information is not precise (small *k*), then, even for a relatively strong reaction (large *α*), the result for the market share is slim (realizations of the Markov chain wander about *x* = 1/2). It takes precise information and a strong reaction in a large population to influence the market share of a given brand for a longer time. Whether this can be achieved in reality is not clear at this time.^13^

## Appendix A

Consider the following differential equation

$$\dot{x} = f(x, \alpha), \quad x \in \mathbb{R}, \alpha \in \mathbb{R}. $$

Following (Wiggins 2003, p.372), in order to show that the above equation undergoes a pitchfork bifurcation at $ (x, \alpha )=(\hat {x}^{},\alpha _{0}) $, it is sufficient to show that

$$\begin{array}{@{}rcl@{}} f(\hat{x}^{},\alpha_{0})& =& 0, \qquad \frac{\partial f}{\partial x} (\hat{x}^{},\alpha_{0})= 0, \\ \frac{\partial f}{\partial \alpha} (\hat{x}^{},\alpha_{0}) &=& 0, \qquad \frac{\partial^{2} f}{\partial x^{2}} (\hat{x}^{},\alpha_{0}) = 0, \\ \frac{\partial^{2} f}{\partial x \partial \alpha} (\hat{x}^{},\alpha_{0}) & \neq & 0, \qquad \frac{\partial^{3} f}{\partial x^{3} } (\hat{x}^{},\alpha_{0}) \neq 0. \end{array} $$

For Eq. 5, we have $ (\hat {x}, \alpha _{0}) = (1/2, 5/6) $ and the following calculations

$$\begin{array}{@{}rcl@{}} f(\hat{x}^{},\alpha_{0})& =&0,\\ \frac{\partial f}{\partial x} (\hat{x}^{},\alpha_{0}) &=& (6 (1-2 \alpha ) (x-1) x-1)|_{(\hat{x}^{},\alpha_{0})}=0, \\ \frac{\partial f}{\partial \alpha} (\hat{x}^{},\alpha_{0}) &=& (-4 x^{3}+6 x^{2}-1)|_{(\hat{x}^{},\alpha_{0})}=0,\\ \frac{\partial^{2} f}{\partial x^{2}} (\hat{x}^{},\alpha_{0}) &=& (-6 (2 \alpha -1) (2 x-1))|_{(\hat{x}^{},\alpha_{0})}=0, \\ \frac{\partial^{2} f}{\partial x \partial \alpha} (\hat{x}^{},\alpha_{0}) & =& (-12 (x-1) x )|_{(\hat{x}^{},\alpha_{0})}=3 \neq 0, \\ \frac{\partial^{3} f}{\partial x^{3} } (\hat{x}^{},\alpha_{0}) & =& (12-24 \alpha )|_{(\hat{x}^{},\alpha_{0})}=-8\neq 0. \end{array} $$

For Eq. 8, we have

$$(\hat{x}, \alpha_{0}) = \left(1/2,\frac{1}{4} \left(\frac{\sqrt{2 \pi }}{\sqrt{k}}+2\right)\right) $$

and the following calculations

$$\begin{array}{@{}rcl@{}} g(\hat{x}^{},\alpha_{0})& =&0,\\ \frac{\partial g}{\partial x} (\hat{x}^{},\alpha_{0})& =& \left(\frac{(2 \alpha -1) \sqrt{k} \mathrm{e}^{\frac{k (1-2 x)^{2}}{8 (x-1) x}}}{4 \sqrt{2 \pi } (-(x-1) x)^{3/2}}-1\right)|_{(\hat{x}^{},\alpha_{0})}=0, \\ \frac{\partial g}{\partial \alpha} (\hat{x}^{},\alpha_{0}) & =& \left(\text{erf}\left(\frac{\sqrt{k} (2 x-1)}{2 \sqrt{2} \sqrt{-(x-1) x}}\right)\right)|_{(\hat{x}^{},\alpha_{0})}=0,\\ \frac{\partial^{2} g}{\partial x^{2}} (\hat{x}^{},\alpha_{0}) & =& \left( -\frac{(2 \alpha -1) \sqrt{k} (2 x-1) \mathrm{e}^{\frac{k (1-2 x)^{2}}{8 (x-1) x}} (k+12 (x-1) x)}{32 \sqrt{2 \pi } (-(x-1) x)^{7/2}} \right)|_{(\hat{x}^{},\alpha_{0})}=0, \\ \frac{\partial^{2} g}{\partial x \partial \alpha} (\hat{x}^{},\alpha_{0}) & =& \left(\frac{\sqrt{k} \mathrm{e}^{\frac{k (1-2 x)^{2}}{8 (x-1) x}}}{2 \sqrt{2 \pi } (-(x-1) x)^{3/2}} \right)|_{(\hat{x}^{},\alpha_{0})}= 2 \sqrt{\frac{2}{\pi }} \sqrt{k} \neq 0, \\ \frac{\partial^{3} g}{\partial x^{3} } (\hat{x}^{},\alpha_{0}) & =& \left(\frac{ (2 \alpha -1) \sqrt{k} \mathrm{e}^{\frac{k (1-2 x)^{2}}{8 (x-1) x}}}{256 \sqrt{2 \pi } (-(x-1) x)^{11/2}} h(x, \alpha) \right)|_{(\hat{x},\alpha_{0})}= -4 (k-3) \neq 0, \end{array} $$

where

$$\begin{array}{@{}rcl@{}} h(x,\alpha) &=& \left(k^{2} (1-2 x)^{2}+8 k (x-1) (18 (x-1) x+5) x \right.\\ &&\left. +48 (x-1)^{2} (16 (x-1) x+5) x^{2}\right). \end{array} $$

## Footnotes

1 The parameter *k* is essentially the sample size. The larger the sample size, the more precise information about the majority in the general population that can be obtained by an individual. For small *k*, there is high probability that the majority within a sample is different than the majority in a general population.

2 This is the standard setting for evolutionary game theory and the theory of learning in population games. In the context of the presented model, the choices of individuals are done when a device being used breaks. For all individuals, these events may be modeled by independent Poisson processes. Thus the probability that two devices break at the same time is zero. Consequently, at any given time, there is only a single individual making a decision.

3 It is assumed that the reference group is selected at random at each time, which is typical for evolutionary game theory. The main reason for this assumption is that it makes the analytical analysis of the model possible. More detailed discussion of this assumption is postponed to Section 3.

4 The thorough explanation of the relation between solutions of the system (1) and realizations of the Markov chain is certainly out of the scope of the present paper; cf. (Benaim and Weibull 2003). Consider the solution of the system (1) and a realization of the Markov chain both starting from the same state. It can be shown that, for any *𝜖* and any time interval, there exists the size of the population *N* large enough that the probability of a realization of the Markov chain deviating from the deterministic solution of the system (1) by more than *𝜖* is arbitrary small. (Cf. (Benaim and Weibull 2003), p. 880, Lemma 1.) The basic intuition behind this result is that, in a single step of the Markov chain, the average change in shares is exactly (*p*_21_(*x*)−*p*_12_(*x*))/*N*. The actual realization in a single step differs from this value (since it is always ±1/*N*) but for large populations, this difference is arbitrary small. If the probabilities *p*_*ij*_(*x*) are continuous in *x*, then the propagation of the difference between the actual realization of the Markov chain and the system of differential equations is mitigated because the values of *p*_*ij*_(*x*) do not change too much if the values of *x* do not change much and, with high probability, a realization of the Markov chain remains in a vicinity of the solution of the deterministic system of differential equations. Consider the realization of the Markov chain starting from a state in a neighborhood of a stable equilibrium of the system (1). It can be shown that the probability of the exit time from the neighborhood being smaller than some time *t* is arbitrary small for the appropriately large size of the population. In other words, the realizations of the Markov chain will stay arbitrary long in a vicinity of a stable equilibrium of the system (1). (Cf. (Benaim and Weibull 2003), p. 895, Lemma 3.) It can be also shown that, for large populations, the Markov chain almost surely spends almost all the time, in the long run, in the vicinity of a set of stable equilibria of the system (1) or, more precisely, in the intersection of the finite (but very dense) grid of states of the Markov chain and the neighborhood of the set of stable equilibria of (1). (Cf. (Benaim and Weibull 2003), p. 884, Proposition 4.) It is much harder to provide a precise intuition behind those results because they require definitions of certain mathematical objects that are beyond the scope of this paper.

5 However, in Section 3, a clear distinction is made between the approximation and the actual Markov chain.

6 For *k* = 1, the model is even simpler but it lacks any interesting features. Any realization of the Markov chain for any *α* < 1 wanders about *x* = 1/2 and increasing *α* leads to increasing volatility. For *k* = 2, the results are identical (the transition probabilities are identical). The value *k* = 3 is the lowest value that produces the behavior observed for all higher values of *k*.

7 The Eq. 5 can be rewritten as

$$\dot{z} = - z \left(z^{2} + \frac{5-6\alpha}{2}\right), $$

where $ z = (x-1/2) \sqrt {4\alpha - 2} $. Formal proof is moved to Appendix A.

8 Formal proof is moved to Appendix A.

9 The differential equation (8) is an approximation, the better the larger the size *N* of the population. It is assumed that *k* < *N*, so technically the limit of *α*_0_ as *k* → *∞* is only possible once the limit *N* → *∞* has been taken. However, in the context of the background story, only relatively small values of *k* are considered and the limit is just provided for completeness.

10 Using formulas from (Benaim and Weibull 2003, p. 895, Lemma 3), we know that the average exit time from the neighborhood of an equilibrium is longer than exp(*γN*)/4−1 for some positive constant *γ*>0 not depending on *N*.

11 The Poisson graph is the type of a random graph introduced in a series of papers (Erdos and Renyi 1959; 1960; 1961).

12 For rules based on the best-reply mapping, a payoff function must associate higher payoffs with the brand used by the majority of a population (leading essentially to the coordination game).

13 There are other questions concerning ethical and legal issues. Also, it is not quite clear if it is possible to manipulate the follow-up probability, despite the claims made in Kramer et al. (2014).
